# Untrained birds’ ability to recognise predators with changed body size and colouration in a field experiment

**DOI:** 10.1186/s12862-021-01807-8

**Published:** 2021-05-01

**Authors:** Kateřina Antonová, Petr Veselý, Roman Fuchs

**Affiliations:** 1 Faculty of Science, Charles University in Prague, Albertov 6, 12800 Praha 2, Czech Republic; 2 Faculty of Science, University of South Bohemia, Branišovská 1760, 37005 České Budějovice, Czech Republic

**Keywords:** Predator recognition, Body size, Colouration, Birds

## Abstract

**Background:**

During recognition process, multiple parameters of the encountered stimulus may play a role. Previous studies with wild birds identified the importance of several salient features (e.g., eyes, beak, prominent elements of colouration) which birds use to recognise other bird species, such as predators or nest parasites. In the present study, we observed the responses of passerines visiting winter feeders to stimuli in the form of dummies of Eurasian sparrowhawk which were modified in body size and/or colouration but always carried the salient features of raptors (hooked beak, talons) and one species-specific feature of the sparrowhawk (yellow eyes). In the vicinity of a feeder, we placed a dummy of an unmodified sparrowhawk, life-sized sparrowhawk with pigeon, great tit, or robin colouration, a small, great tit-sized sparrowhawk dummy with unmodified or pigeon colouration, or an unmodified pigeon dummy, which functioned as a harmless control. Then we measured how it affected the number of visits.

**Results:**

We found that birds were less afraid of small dummies regardless of their colouration than they were of life-sized raptor dummies or even the pigeon dummy. This contrasts with the results of a previous laboratory experiment where great tits’ reaction to small dummies was comparably fearful to their response to life-size dummies. In our experiment, birds were also not afraid of life-sized dummies with modified colouration except for a robin-coloured dummy, which caused an equally significant fear reaction as an unmodified sparrowhawk dummy. It is likely that this dummy resembled the colouration of a male sparrowhawk closely enough to cause this effect.

**Conclusions:**

Based on our observations, we conclude that birds use contextual features to evaluate the size of other birds. Distance and familiar reference points seem to play an important part in this process.

**Supplementary Information:**

The online version contains supplementary material available at 10.1186/s12862-021-01807-8.

## Background

Every animal has the ability to recognise various objects relevant to its life, including other animals such as conspecifics, competitors, prey, or predators. In all animals which are preyed upon, the ability to reliably recognise predators is particularly important because it is crucial to an adequate antipredator response and therefore also their immediate survival [1].

Recognition process sensu Cohen and Lefebvre [2] consists of discrimination, i.e., the ability to tell apart one object from the other, and categorisation, that is, inclusion of an object into a category of other objects based on their similarity. In the case of predators, it is essential for their potential prey to distinguish them from harmless animals and place them in the appropriate category. Predators can be categorised on different levels: predators in general or groups of predators with common characteristics, such as diurnal aerial predators (raptors), nocturnal aerial predators (owls), ground predators (carnivorans), or particular predator species. For instance, both the Eurasian sparrowhawk (*Accipiter nisus*) and Eurasian kestrel (*Falco tinnunculus*) are small raptors, but they pose different levels of threat to passerine birds [3]. In this case, proper species recognition on the part of their prey prevents an inappropriate response.

The study of predator recognition started with a number of classic works of ethology [4–6], which found that in raptor recognition, birds use simple sub-features. In Lorenz’s terminology, they are called ‘releasers’ (*Auslöser*), in Tinberger’s approach, they are called ‘sign stimuli’. In the abovementioned studies, representative of such features was for instance the short neck and long tail of the raptor silhouette. Subsequent studies demonstrated that birds distinguish raptors from harmless birds mainly based on the presence of the former’s hooked beak (e.g. [7–10]), but further evidence indicates that birds also identify particular raptor species by characteristic elements of colouration [11–15].

The Eurasian sparrowhawk, a specialised predator of small passerines [16, 17], is commonly used in studies that test the recognition of predators (see [18] for review). As noted above, there is some evidence to the effect that birds can distinguish the sparrowhawk from other small raptors, such as the Eurasian kestrel, by horizontal undulating stripes on the belly and conspicuous yellow eyes [19–21]. The cuckoo-hawk mimicry hypothesis, which suggests that the grey morph of the parasitic common cuckoo (*Cuculus canorus*) mimics the sparrowhawk, further supports the importance of this colouration feature [22].

Hooked beak and prominent talons, as well as species-specific components of colouration, seem to act as salient features sensu Tinbergen [6] and a number of experiments (see above) support this hypothesis. On the other hand, these features are only part of the various traits that characterise any raptor. For example, all raptor species have a body plan with a specific and distinctive mutual position and proportion of the body parts. In the Eurasian sparrowhawk specifically, a conspicuously horizontally striped belly and yellow eyes are complemented by slate-grey or brownish upper parts and wings.

The other general characteristic of each species is its body size. It is undoubtedly important for predator recognition because it is frequently linked to their specialisation in terms of prey or diet in general (e.g. [23]). Templeton et al. [24] demonstrated that the alarm calls of black-capped chickadee (*Poecile atricapilla*) vary with the size of the encountered predator. Galliformes, too, perform different antipredator behaviour in response to predators of different body size [25]. It seems therefore that birds perceive predator size and use it as an important cue for threat assessment. What is yet to be determined is the relative importance of body size in the context of other features characteristic of particular predator species.

Beránková et al. [26] observed the ability of untrained great tits (*Parus major*) in laboratory conditions (in an indoor cage) to recognise dummies that carried the salient features of raptors (hooked beak, talons) and one of the species-specific features of the Eurasian sparrowhawk (yellow eyes). They modified dummies’ size and colouration. In one part of the experiment, they reduced the body size of a male Eurasian sparrowhawk (app. 32 cm) to great tit size (app. 16 cm), while retaining the salient features in their original size. One dummy had unmodified sparrowhawk colouration with the typical horizontal stripes on the belly. The other three dummies had the colouration of birds harmless to great tits, namely that of domestic pigeon (*Columba livia* f. *domestica*), European robin (*Erithacus rubecula*), and great tit. The most important result of the study was the finding that dummies with sparrowhawk colouration evoked fear regardless of their size. In the experiment, the great tits were in close contact with the predator dummy, because it was presented in front of their two-metre-long cage. Moreover, the tits could not directly compare the size of the presented dummy with any familiar object. On the other hand, great tit-coloured dummies evoked no fear reaction regardless of their size. This indicated that colouration may be more important than size, although dummy size was more important in robin-coloured dummies and, although less strongly, size also played a role in great tits’ reaction to the pigeon-coloured dummies. The authors thus concluded that in species less important to the tested birds (and perhaps also in birds which they recognise less readily), size is important. When, however, colouration represents a predator or a conspecific, the importance of size is reduced.

**Fig. 1:**
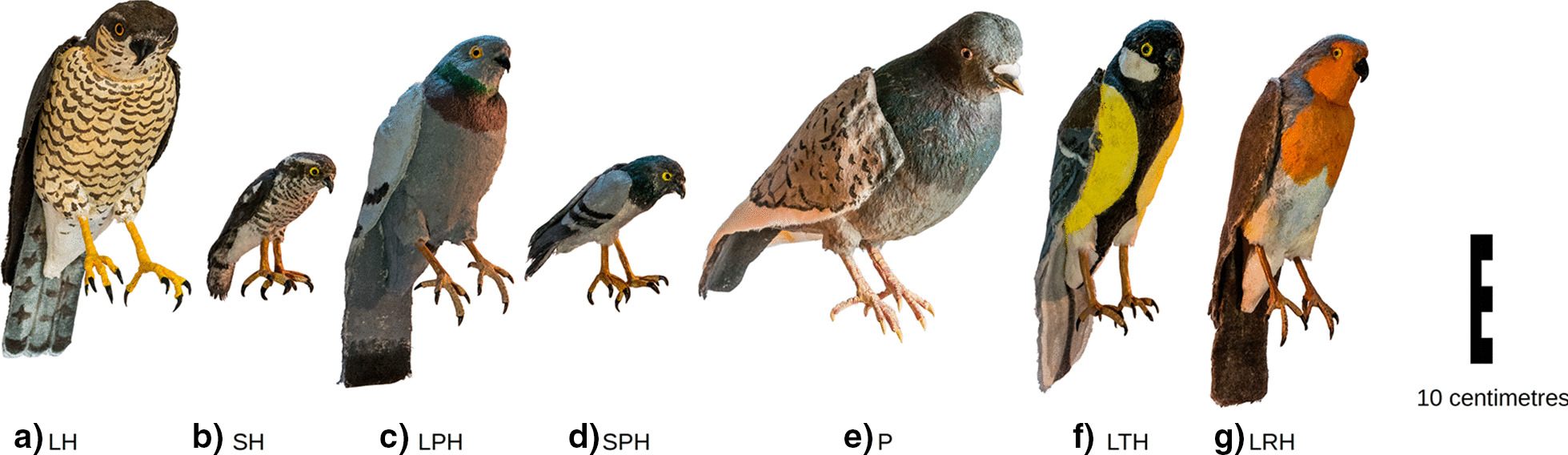
Dummies presented in the experiments. **a** Unmodified sparrowhawk (‘large hawk’ LH), **b** downsized sparrowhawk with unmodified colouration (‘small hawk’ SH), **c** life-sized sparrowhawk with pigeon colouration (‘large pigeon-hawk’ LPH), **d** downsized sparrowhawk with pigeon colouration (‘small pigeon-hawk’ SPH), **e** unmodified domestic pigeon (‘pigeon’ P), **f** life-sized sparrowhawk with robin colouration (‘large robin-hawk’ LRH) and **g** life-sized sparrowhawk with great tit colouration (‘large tit-hawk’ LTH)

We decided to repeat this experiment in field conditions with dummies presented at winter feeders visited by wild birds. The birds could compare the presented dummies with other objects, including other birds near the feeder. Such field experiments are more time-consuming than experiments in laboratory conditions, which is why we split it in two parts. In the first experiment, we tested four dummies: an unmodified sparrowhawk dummy (body length 32 cm), a downsized sparrowhawk dummy (body length 16 cm) with unmodified colouration, a life-sized sparrowhawk dummy with pigeon colouration, and a downsized sparrowhawk with pigeon colouration (see Fig. 1). In the second experiment, we worked with three sparrowhawk dummies: an unmodified sparrowhawk dummy, a life-sized sparrowhawk dummy with tit colouration, and a life-sized sparrowhawk dummy with robin colouration (see Fig. 1).

Experiments were conducted in the mode of a two-feeder arrangement [13, 27]). The tested birds could see both feeders simultaneously and choose which, if any, to visit. At each feeder, we placed a different dummy. One was in most cases an unmodified, i.e., life-sized and naturally coloured dummy of a sparrowhawk or domestic pigeon (a harmless bird with size comparable to the sparrowhawk, Fig. 1), while the other dummy was one of the modified ones (see above). Use of the pigeon dummy allowed us to eliminate the effect of potential food competition on the feeder on the process of predator recognition. The two-feeder arrangement design allowed for a more subtle evaluation of bird behaviour than a straightforward consecutive presentation of dummies at a single feeder would [13].

### We tested the following hypotheses

#### Null hypothesis

Birds recognise all modified dummies as a raptor (i.e., they are more afraid of them than they are of the dummy of a harmless domestic pigeon) or a sparrowhawk (i.e., they are at least as afraid of them as they are of the unmodified sparrowhawk dummy). Salient features (hooked beak, talons, yellow eyes) are more important for recognition than colouration or body size.

#### Alternative hypotheses

AH 1: Birds recognise only life-sized dummies as a raptor or a sparrowhawk.

AH 2: Birds recognise dummies with modified colouration as a raptor but not as a sparrowhawk (yellow eyes are not sufficient for recognising the dummy as a sparrowhawk).

AH 3: Birds do not recognise dummies with modified colouration as either a raptor or a sparrowhawk (a hooked beak, talons, and yellow eyes do not suffice for the dummy to be recognised as a raptor).

## Results

### First experiment

In the first experiment, the dummy at the focal feeder (i.e. the feeder analysed at the moment), the species of bird landing at the feeder, interaction between dummies at the focal and non-focal feeder, and interaction between dummy at the focal feeder and bird species had a statistically significant effect (p < 0.05) on the relative change in the number of birds visiting a feeder (Table 1). We calculated this change as a ratio between the number of birds landing at a feeder during the presentation of a particular dummy and the immediately preceding ‘empty control’, i.e., the number of birds which visited the feeder when no dummy was present.

**Table 1:** Results of linear mixed-effect models

Experiment	Response variable	Predictor variable	df	chi	p
First	Proportion of visits	Focal dummy	4	35.021	** < < 0.001**
		Non-focal dummy	4	7.829	0.098
		Focal*non-focal	17	53.567	** < 0.001**
		Bird species	4	28.644	** < < 0.001**
		Focal*species	16	40.389	** < 0.001**
		Preceding dummy	5	7.896	0.162
		Feeder ID	1	2.548	0.130
		Order	7	9.700	0.206
		Temperature	1	1.515	0.218
		Snow	1	1.906	0.167
Second	Proportion of visits	Focal dummy	3	7.378	0.061
		Non-focal dummy	3	3.657	0.301
		Focal*non-focal	5	35.557	**0.035**
		Bird species	4	24.451	** < 0.001**
		Focal*species	12	17.332	0.138
		Preceding dummy	4	2.551	0.636
		Feeder ID	1	0.071	0.790
		Order	1	3.066	0.080
		Temperature	1	0.824	0.364
		Snow	1	0.087	0.766

Df refers to the degrees of freedom

Asterisk indicates interaction of predictors

Significant effects are in bold

The effect of the type of dummy on the non-focal feeder, i.e. the feeder adjacent to one we focused on, was not significant (Table 1). Other tested variables likewise had no effect on the number of birds visiting a feeder (Table 1).

**Fig. 2:**
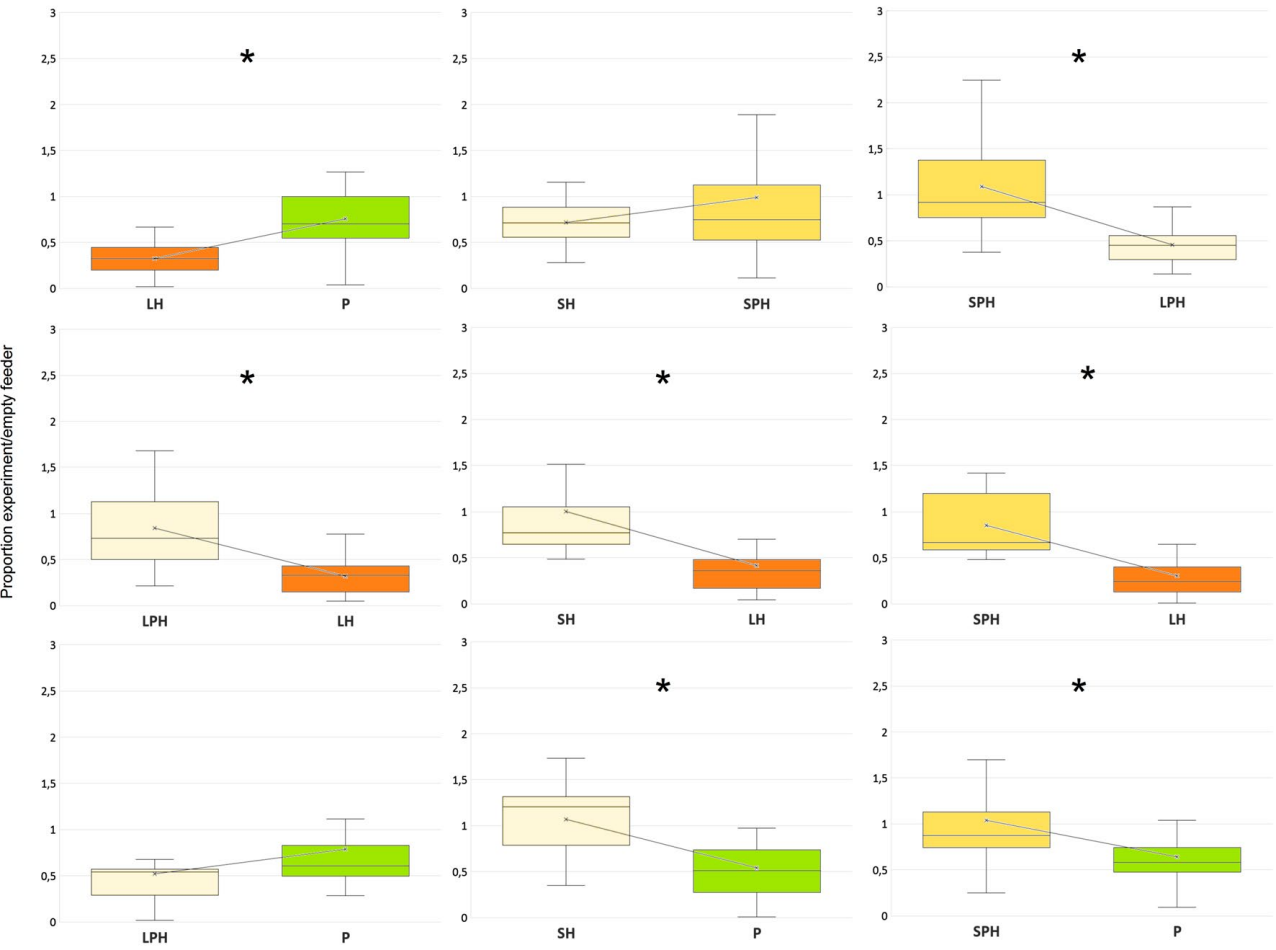
The number of landings of all tested bird species to particular dummy pairs in the first experiment divided by the number of landings during the preceding empty controls. Values below one indicate dummy’s negative effect on landing rate. Asterisk indicates a significant difference (post hoc Tukey HSD t-test). LH = unmodified sparrowhawk (‘large hawk’), P = unmodified domestic pigeon (‘pigeon’), SH = downsized sparrowhawk with unmodified colouration (‘small hawk’), LPH = life-sized sparrowhawk with pigeon colouration (‘large pigeon-hawk’), SPH = downsized sparrowhawk with pigeon colouration (‘small pigeon-hawk’)

A post hoc test of simultaneously tested pairs of dummies showed that the relative decrease in the number of birds visiting feeders with both downsized dummies (downsized sparrowhawk and downsized pigeon-coloured sparrowhawk) was smaller than the relative decrease in the number of visits to feeders with any of the large dummies (pigeon, life-sized sparrowhawk, life-sized pigeon-coloured sparrowhawk; Table 2, Fig. 2).

**Table 2:** The results of parametric pairwise post hoc comparisons (Tukey HSD post hoc tests) of the proportion of visits to feeders with particular dummies in the first experiment

Dummy	LH	SH	LPH	SPH	P
LH	–	− **10.877**	− **14.887**	− **5.230**	− **7.251**
SH	**0.003**	–	–	− 1.727	**13.286**
LPH	** < 0.001**	–	–	− **6.968**	− 2.011
SPH	**0.029**	0.199	**0.013**	–	**4.666**
P	**0.012**	**0.001**	0.167	**0.040**	–

The right-top part of the table refers to t-values, the left-bottom part to p-values. Positivity of t-values indicates that the mean value of the number of visits to a feeder with the dummy in a column is higher than the mean value related to the dummy in the row. LH = unmodified sparrowhawk (‘large hawk’); P = unmodified domestic pigeon (‘pigeon’); SH = downsized sparrowhawk with unmodified colouration (‘small hawk’); LPH = life-sized sparrowhawk with pigeon colouration (‘large pigeon-hawk’); SPH = downsized sparrowhawk with pigeon colouration (‘small pigeon-hawk’)

Significant effects are in bold

The relative decrease in the number of visits to feeder with the life-sized pigeon-coloured sparrowhawk dummy was smaller than the relative decrease in the number of visits to feeder with the unmodified sparrowhawk dummy. In fact, the life-sized pigeon-coloured sparrowhawk dummy caused a similar relative decrease in the number of visits to feeder as the unmodified pigeon dummy did (Table 2, Fig. 2). The relative decrease in the number of visits to feeder with the downsized sparrowhawk dummy with unmodified colouration and visits to feeder with downsized pigeon-coloured sparrowhawk dummy was likewise identical (Table 2, Fig. 2). On the other hand, the relative decrease in the number of birds visits to feeder with the pigeon dummy was, not surprisingly, smaller than the relative decrease in the number of birds visiting a feeder with the unmodified sparrowhawk dummy (Table 2, Fig. 2).

**Fig. 3:**
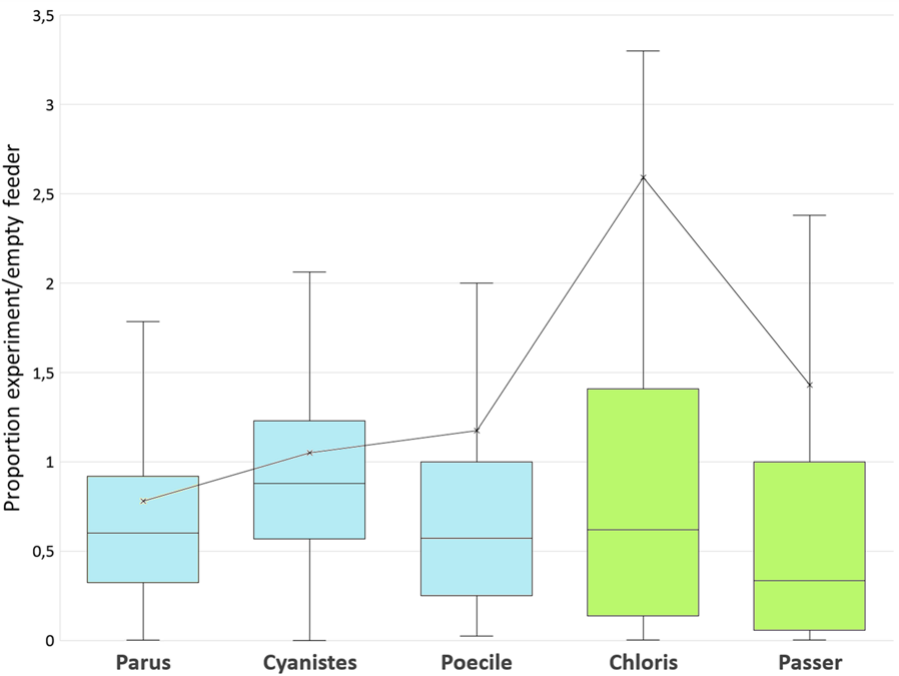
The number of landings of particular bird species to all tested dummies in the first experiment divided by the number of landings during the preceding empty controls. Values below one indicate dummy’s negative effect on the landing rate. Parus—great tit (*Parus major*), Cyanistes—blue tit (*Cyanistes caeruleus*), Poecile—marsh (*Poecile palustris*) and willow tit (*Poecile montanus*) together, Chloris—green finch (*Chloris chloris*), Passer—domestic (*Passer domesticus*) and tree sparrow (*Passer montanus*) together. Titmice taking only a single seed item are in blue, while specialized granivores collecting multiple seeds and thus staying longer at the feeder are in green

Post hoc tests comparing the proportion of birds of particular species visiting a feeder showed that the relative decrease in the number of sparrows was greater than the relative decrease in the number of visits by any of the three tit species (Table 3, Fig. 3).

**Table 3:** Results of parametric pairwise post hoc comparisons (Tukey HSD post hoc tests) of the proportion of visits by particular bird species to the presented dummies in the first experiment

Species	Great tit	Blue tit	Marsh tit	Sparrows	Greenfinch
Great tit	–	− 0.805	− 0.179	− **4.068**	0.873
Blue tit	0.229	–	− 1.374	− **4.395**	− 2.068
Marsh tit	0.764	0.196	−	**4.021**	0.694
Sparrows	** < 0.001**	** < 0.001**	** < 0.001**	–	− 2.673
Greenfinch	0.206	0.090	0.421	0.051	–

The right-top part of the table refers to t-values, the left-bottom part to p-values. Positivity of t-values indicates that the mean value of the number of visits by particular species in a column is higher than the mean value related to the species in the row

Significant effects are in bold

The relative decrease in greenfinch visits was slightly higher than the relative decrease in blue tit visits (Table 3, Fig. 3) but lower than the relative decrease in sparrow visits (Table 3, Fig. 3). The remaining interspecific differences were not statistically significant (Table 3, Fig. 3).

### Second experiment

In the second experiment, the dummy on the focal feeder and the order of tested dummies had a slight effect on the relative change in the number of bird visits, i.e., the ratio between the number of birds landing at a feeder during the presentation of a particular dummy and during the preceding empty control (Table 1). The effect of the species of birds visiting a feeder and interaction of dummies at the focal and non-focal feeder was significant, while other tested variables had no effect on the change in numbers of visiting birds (Table 1).

**Fig. 4:**
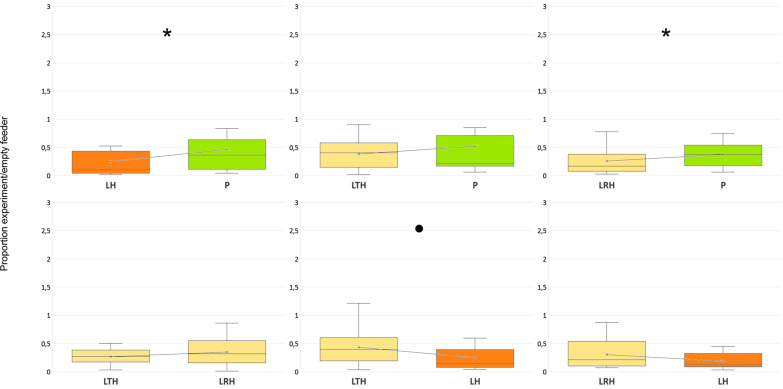
The number of landings of all tested birds to particular dummy pairs in the second experiment divided by the number of landings during the preceding empty controls. Values below one indicate dummy’s negative effect on the landing rate. Asterisk indicates a significant difference; dot indicates a merely indicative level of significance (post hoc Tukey HSD t-test). LH = unmodified sparrowhawk (‘large hawk’), P = unmodified domestic pigeon (‘pigeon’), LTH = life-sized sparrowhawk with great tit colouration (‘large tit-hawk’), LRH = life-sized sparrowhawk with robin colouration (‘large robin-hawk’)

A post hoc test of simultaneously tested pairs of dummies showed that the relative decrease in the number of birds visiting a feeder with the life-sized great tit-coloured sparrowhawk dummy was slightly smaller than the relative decrease in the number of bird visits to feeder with the unmodified sparrowhawk dummy and did not differ from the relative decrease in the number of birds’ visits to feeder with the unmodified pigeon dummy (Table 4, Fig. 4).

**Table 4:** Results of parametric pairwise post hoc comparisons (Tukey HSD post hoc tests) of the proportion of visits to feeders with particular dummies in the second experiment

Dummy	LH	LTH	LRH	P
LH	–	− 3.808	− 2.194	− **5.040**
LTH	0.053	–	− 1.393	− 1.894
LRH	0.141	0.240	–	− **5.584**
P	**0.026**	0.171	**0.020**	–

The right-top part of the table refers to t-values, the left-bottom part to p-values. Positivity of t-values indicates that the mean value of the number of birds’ visits at feeder with the dummy in a column is higher than the mean value related to the dummy in the row. LH = unmodified sparrowhawk dummy (‘large hawk’); P = unmodified domestic pigeon dummy (‘pigeon’); LTH = life-sized sparrowhawk dummy with great tit colouration (‘large tit-hawk’); LRH = life-sized sparrowhawk dummy with robin colouration (‘large robin-hawk’)

Significant effects are in bold

The relative decrease in the number of visits to feeder with the life-sized robin-coloured sparrowhawk dummy was significantly greater than the relative decrease in the number of birds’ visits at feeder with the pigeon dummy and of the same size as the relative decrease in the number of birds’ visits to feeder with the unmodified sparrowhawk dummy (Table 4, Fig. 4). The relative decrease in the number of visits to feeder with the life-sized sparrowhawk robin-coloured dummy was, moreover, of the same size as the relative decrease in the number of birds’ visits to feeder with the life-sized great tit-coloured sparrowhawk dummy (Table 4, Fig. 4). Not surprisingly, the relative decrease in the number of birds’ visits to feeder with the pigeon dummy was smaller than the relative decrease in the number of birds’ visits to feeder with the unmodified sparrowhawk dummy (Table 4, Fig. 4).

**Fig. 5:**
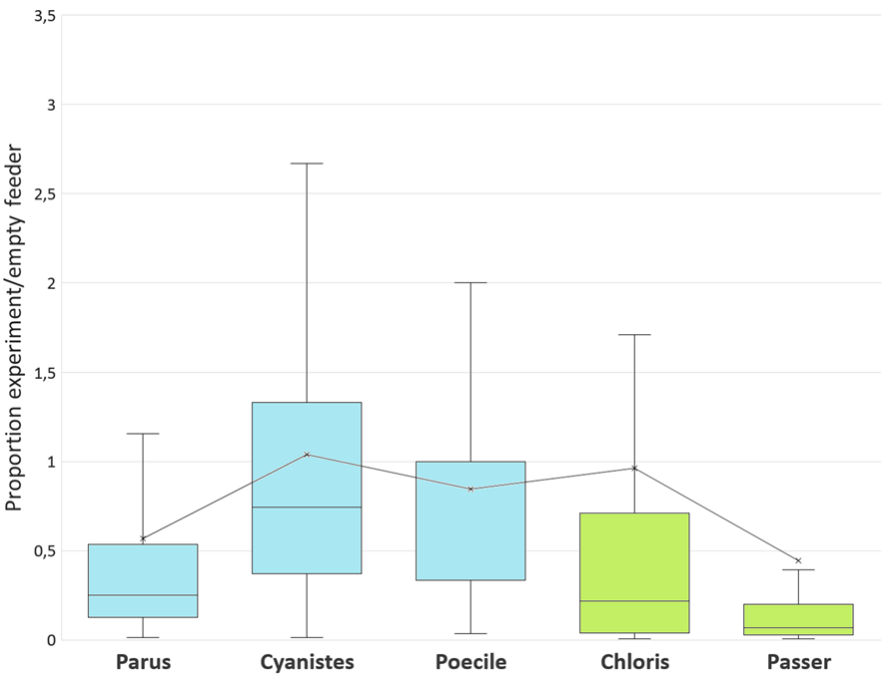
The number of landings of particular bird species to all tested dummies in the first experiment divided by the number of landings during the preceding empty controls. Values below one indicate dummy’s negative effect on the landing rate. Parus—great tit (*Parus major*), Cyanistes—blue tit (*Cyanistes caeruleus*), Poecile—marsh (*Poecile palustris*) and willow tit (*Poecile montanus*) together, Chloris—green finch (*Chloris chloris*), Passer—domestic (*Passer domesticus*) and tree sparrow (*Passer montanus*) together. Titmice taking only a single seed item are in blue, while specialized granivores collecting multiple seeds and thus staying longer at the feeder are in green

A post hoc test comparing the tested species showed that the relative decrease in the number of visits by sparrows was greater than the relative decrease in the number of visits by blue tits (Table 5, Fig. 5).

**Table 5:** Results of parametric pairwise post hoc comparisons (Tukey HSD post hoc tests) of the proportion of visits by particular bird species to feeders with presented dummies in the second experiment

Species	Great tit	Blue tit		Marsh tit	Sparrows	Greenfinch
Great tit	–	− 2.407	1.926		1.070	− 1.280
Blue tit	0.113	–	**4.333**		**3.477**	1.120
Marsh tit	0.303	** < 0.001**	–		− 0.856	− **3.201**
Sparrows	0.822	**0.005**	0.913		–	− 2.347
Greenfinch	0.703	0.796	**0.012**		0.130	–

The right-top part of the table refers to t-values, the left-bottom part to p-values. Positivity of t-values indicates that the mean value of the number of visits by particular species in a column is higher than the mean value related to the species in the row

Significant effects are in bold

The relative decrease in the number of visits by marsh tits was greater than the relative decrease in the number of visits by greenfinches and blue tits (Table 5, Fig. 5). The remaining interspecific differences were not significant (Table 5, Fig. 5).

## Discussion

In the first experiment, passerines visited feeders with modified dummies (life-sized pigeon-coloured sparrowhawk, downsized sparrowhawk, downsized pigeon-coloured sparrowhawk) more often than they visited the feeder with unmodified sparrowhawk dummy. This supports the hypothesis that the birds did not perceive any of the modified dummies as a sparrowhawk. Furthermore, birds were visiting feeders with both of the downsized dummies more frequently than they did on the feeder with an unmodified pigeon dummy and they visited the feeder with the life-sized pigeon-coloured sparrowhawk dummy as often as they did the feeder with pigeon dummy. This seems to indicate that the birds did not recognise any of the modified dummies as a raptor either.

In the second experiment, birds visited the feeder with a life-sized robin-coloured sparrowhawk dummy as often as they did the feeder with life-sized sparrowhawk dummy and less than the feeder with the pigeon dummy. This indicates that the birds perceived the large robin-coloured sparrowhawk dummy as a raptor and most likely even as a sparrowhawk. On the other hand, birds visited the feeder with life-sized great tit-coloured sparrowhawk dummy more often than the feeder with the unmodified sparrowhawk dummy and as often as the feeder with the pigeon dummy. It indicates that the birds did not recognise the with life-sized sparrowhawk dummy with great tit colouration as either a raptor or a sparrowhawk.

Based on the results of both experiments, it seems that the presence of salient features alone is not sufficient for proper recognition of either a sparrowhawk or, more generally, a raptor.

Our first experiment strongly indicates that size plays a crucial role in the recognition of predators. Our findings suggest that birds did not recognise the downsized dummies as a threat although they had not only the salient sparrowhawk features but also its colouration.

Our field experiments follow upon the laboratory cage experiment of Beránková et al. [26]. In their experiment, but not ours, great tits showed a similar intensity of fearful behaviour when presented with a large or small dummy with either sparrowhawk or pigeon colouration. This discrepancy in findings might be due to differences in the experimental setup. In the laboratory experiment, the great tits were close to the dummy, which allowed them a detailed inspection of its salient features (yellow eyes, talons, hooked beak). Moreover, there were no familiar objects close by which the birds could use to assess the size of the dummies. In our experiments, on the other hand, the birds landing at feeders could inspect the dummy from a much greater distance, so the dummy’s size—which they could assess based on comparison with a variety of nearby familiar objects (vegetation, other birds)—could serve as the first clue used in evaluating a potential threat. And indeed, whenever the dummy was significantly smaller than any potential raptor, most birds paid no attention to it.

The experiment which compared reactions to two downsized dummies (downsized sparrowhawk vs. downsized pigeon-coloured sparrowhawk) supports this hypothesis. The parametric test showed no significant difference between visiting rates to feeders with the two dummies, while the nonparametric Mann–Whitney (Additional file 3: Tables S7, S8) comparisons did reveal a difference. It indicates that the size of relative change of numbers of birds visiting feeders with either of the small dummies is similar but in most trials, the relative decrease in visiting rates was greater for the feeder with sparrowhawk dummy with unmodified colouration. This could be interpreted as meaning that at least some birds perceived the downsized dummy with unmodified colouration as a threat.

Our previous research suggests that individual birds react to dummies with conflicting features in different ways [21, 26]. Personality could play an important role in the process of recognition of such stimuli [28] but the level of individual experience with real sparrowhawks is also likely to affect individual birds’ reactions. For the anonymous wild population we observed, neither of these parameters could be detected or measured.

It has repeatedly been demonstrated that various bird species can distinguish between raptors differing in body size. Palleroni et al. [25] presented three species of trained live raptors from the genus *Accipiter* importantly differing in their body size to domestic hens (*Gallus gallus* f. *domestica*). Hens showed more fear in the presence of the larger, and therefore more threatening, species. Similarly, analysis of variability in warning calls showed that wild Carolina chickadees (*Poecile carolinensis*), tufted titmice (*Baeolophus bicolor*), and captive black-capped chickadees respond differently to stuffed raptors of various sizes. This seems to reflect their perception of a different degree of potential threat these raptors might present [24, 29, 30]. On the other hand, all of these abovementioned studies worked with real predators, which along with variation in size provide other features that can be used for recognition. We provide evidence that size itself is important for the assessment of potential threat, but we also show that birds need further (environmental) information to determine the size of a potentially threatening object.

Our conclusions find further support in studies that used simplified silhouettes of flying raptors. It has been shown that domestic chickens as well as blue tits recognise differences in their size and are more afraid of larger silhouettes than of the smaller ones [31, 32]. Because the silhouettes did not represent any specific identifiable species, the authors assumed that larger silhouettes were simply evaluated as a predator that is closer. Similarly to Beránková et al. [26], however, these results show that birds need further information, such as species-specific colouration or other objects with known size, to determine the magnitude of the potential threat.

There is moreover evidence that birds take size into account even in the case of recognition of artificial objects. Pigeons performed worse when asked to discriminate between artificial objects presented at different sizes than those which were used in training (e.g. [33–38]). Moreover, pigeons seem to be better at recognising simple 2D objects (mostly simple geometric shapes, in some cases line drawings of complex objects) when those objects are large or larger than those they are accustomed to by training than those which are smaller than what they are used to [33, 34, 37]. These results were confirmed by Peissig et al. [37] also in the case of a simple 3D object (barrel). Peissig and colleagues account for this by claiming that pigeons’ ability to recognise basic geometric shapes improves with increased size of the object. They also admit, however, that this is surprising because pigeons are perfectly capable of identifying small seeds that form most of their natural diet. Our results provide another explanation for a similar phenomenon. It is likely that birds on feeders did not identify the downsized raptor dummies as a threat because small raptors simply do not represent a threat so there is no need to pay them much attention. A similar explanation could be applied to the recognition of artificial complex objects, including the stimuli tested by Peisssig et al. [37].

Based on the results of the second experiment, we cannot establish any uniform effect of colouration on the recognition of presented stimuli. The birds did not perceive the life-sized dummies with great tit or pigeon colouration as a threat, but they treated the sparrowhawk dummy with robin colouration as a threat on a level comparable to the unmodified sparrowhawk. None of the imitated species (domestic pigeon, great tit, or European robin) present any danger to small passerines and all of the tested passerine species in our experimental locality commonly encounter the species whose colouration we imitated. Great tits are the most abundant visitors to our feeders, so the tested birds were probably more familiar with their colouration than they were with pigeons or robins. On the other hand, the pigeon-coloured sparrowhawk dummy was of an appropriate size (since pigeons and sparrowhawks are of highly similar size), while the dummies with robin and great tit colouration were much larger than real robins or great tits, which could make their recognition more difficult. Moreover, the robin’s orange breast could seem similar to the underparts of sparrowhawk males, which are sometimes reddish-brown.

In contrast to our first experiment, the second experiment produced similar results to the cage experiments of Beránková et al. [26]. In the cage, great tits exhibited intensive fear behaviour in the presence of a life-sized robin-coloured dummy while life-sized dummies with great tit and pigeon colouration were not perceived as a threat. Our results are entirely consistent with these findings.

Veselý et al. [15] also manipulated sparrowhawk colouration in a winter feeder experiment. Great tits and blue tits on the feeder seemed to perceive a dummy which lacked a typical sparrowhawk colour feature (the undulating stripes across the belly) and a robin-coloured dummy as being as dangerous as an unmodified sparrowhawk dummy. On the other hand, dummies with fully artificial violet-and-white chequered colouration were not perceived as threatening at all. In our experiments, too, only obviously non-raptor colouration (pigeon, great tit) managed to override all of the general salient raptor features of a dummy.

The importance of colouration is further confirmed by studies that tested the cuckoo-hawk mimicry hypothesis [22] which assumes that a horizontally striped belly and yellow eyes protect the grey morph of the cuckoo against an aggressive reaction of its host passerines. In Welbergen and Davies’s study [39], reed warblers (*Acrocephalus scirpaceus*) approached a cuckoo whose undulating horizontal stripes on the belly were removed up to a closer distance than they did an unmodified cuckoo dummy. The reed warblers also kept a greater distance when the horizontally striped belly was painted on a dummy of the Eurasian collared dove (*Streptopelia decaocto*). In a study by Trnka and colleagues [20], on the other hand, great reed warblers (*Acrocephalus arundinaceus*) mobbed a cuckoo with black eyes significantly less than an unmodified cuckoo, while a removal of the belly pattern resulted in no such drop in the number of attacks. Overall, it seems that in these experiments, the warblers were more sensitive to changes in colouration than the birds in our feeder experiments. It might be due to the fact that birds adjust their nest defence to actual threat but at a feeder, they tend to take precautions [18].

Our results point to some potential weaknesses inherent in feeder experiments. In both experiments, the pigeon dummy led to a big drop in the number of birds visiting a feeder. Based on previous studies [12, 13, 15, 40, 41], we chose a pigeon as a harmless control of sparrowhawk size, but it was not perceived as such, that is, as harmless. Of course, pigeons are not predators, but they can act as potentially strong food competitors on the feeder. Generally, our experimental locality seems rich in wintering birds. We counted on average 220 birds visiting one feeder during the empty controls, which in itself indicates strong competition and has no analogy in previous experiments [12, 13, 15, 40]. Such high competition could help account for the fact that in our experiment, birds evinced a stronger avoidance of the pigeon dummy than in previous studies. Grim [42] believes that pigeons, and doves in general, should not be used as harmless control stimuli because some passerines do not view them as such. On the other hand, there is no other way to prove that birds discriminate between predators and ‘non-predatory birds’ of a similar size in field experiments [18].

In the second experiment, we saw a generally lower feeder attendance. The winter of 2017/2018, when the observations took place, was characterised by generally colder temperatures and we also detected on the feeder some bird visitors from the north, namely bramblings (*Fringilla montifringilla*). Increased presence of this species might imply the presence of different populations of other species, especially the tits [43–45]. Such new populations may have likewise arrived from the north and be more tolerant of cold weather, search for food elsewhere, and possibly feel no need to visit feeders where there is any risk or competitors.

The relative change in the number of visiting birds varied considerably among the studied species. European greenfinches and the sparrows were more cautious, which is most likely the result of their foraging strategy. They feed in a group and tend stay on a feeder for a longer time than tits do. They are thus more exposed to predation, which makes them more cautious [46, 47]. Moreover, they are highly efficient at opening the sunflower seeds [48], which means that they can feed fast and do not need to visit the feeder repeatedly. Tits, on the other hand, take a particular seed to the shelter of shrubs, where they open and eat it, so they do need to visit the feeder repeatedly. This could account for why they took bigger risks and did not wait for optimal conditions. Among tits, the blue tits risked more than the great tits. This may be due to their smaller size, which could imply poorer competition ability but also greater nimbleness [49, 50].

## Conclusion

We conclude that presence of salient features (hooked beak, talons, yellow eyes) on their own was insufficient for the recognition of a dummy as the sparrowhawk or a raptor. Our results suggest that predator recognition depends also on the presence of other contextual features, such as colouration and size. The effect of changing dummy size was particularly surprising. Birds were not afraid of downsized dummies regardless of their colouration, even if the dummy was one of a downsized sparrowhawk with unmodified colouration and salient features. Birds also were not afraid of life-sized dummies with modified colouration except for the robin-coloured dummy.

Our results indicate that birds assign ‘novel objects’, in our case artificially created dummies, to categories of real birds. Life-sized sparrowhawk with robin colouration resembles—due to certain features, especially a reddish belly—a sparrowhawk. Life-sized sparrowhawks with pigeon and great tit colouration resemble more closely the respective harmless birds, despite the presence of salient raptor features (hooked beak, talons, yellow eyes).

Recognition and evaluation of predators also seem to depend on the environment of the encounter. Birds reacted in different ways in natural and laboratory conditions. In the wild, they assessed potential predators largely based on their size. In laboratory conditions, on the other hand, size did not play a role. This may suggest that birds assess size based on other common objects present in the context. The results of our field experiments are not so straightforward in cases where birds had to evaluate contradictory features of life-sized dummies: in those cases, it seems that individual birds categorised these dummies in different ways.

## Methods

### Experimental site and species

The experiments were conducted over three winters (2015/2016, 2016/2017, and 2017/2018) in an old orchard near the village of Krupá, central Bohemia, Czech Republic (50.0177594 N, 14.8818522 E). The site is 300 m above sea level and located near a small stream and floodplain forest. We have reasons to believe that this site is a corridor for local as well as migrating birds. We focused on seven species of small passerines most abundant at local feeders: four species of tit (classified in three groups), the European greenfinch, and two species of sparrows (classified as one species). Of the tit species, we observed the great tit (*Parus major*), the blue tit (*Cyanistes caeruleus*)*,* and a ‘marsh’ tit. The latter label actually represented two species, the marsh tit (*Poecile palustris*) and the willow tit (*Poecile montanus*), but since these two species were indistinguishable in the video recording, we treated them as one. This was a simplification that had no bearing on our results. Then, as noted above, we observed the European greenfinch (*Chloris chloris*) and ‘sparrows’. This label once again contained two species, the house sparrow (*Passer domesticus*) and Eurasian tree sparrow (*Passer montanus*). The two sparrow species visited feeders in mixed flocks and precise identification was not always possible. For this reason, we decided to treat the two species in our analyses together as ‘sparrows’.

Neither this nor the second experiment involved any manual handling or invasive manipulation with the birds. Each bird remained in its natural environment during and after the experiment and was allowed to freely move its natural area.

We placed two feeders in a small clearing surrounded by rose hip bushes (*Rosa canina*) and young English oak trees (*Quercus robur*). The shortest distance from cover to feeder was one meter. The two feeders were 20 m apart and the space between them was devoid of woody plants. From their shelter in the shrubs, the birds could see both feeders at the same time. The feeders were made of a wooden frame (70 × 70 cm) placed on the ground (for a scheme of experimental site, see Additional file 4).

As fodder for the birds, we used sunflower seeds. The studied birds used two foraging strategies: Tits landed at the feeder, took one seed, and flew to a shelter to process it. Finches and sparrows, on the other hand, would come to the feeder and start feeding continuously without leaving the feeding spot. At first sight, these two strategies differ substantially in the level of risk the birds expose themselves to. It may seem that by staying for a long time at the exposed feeder, the finches and sparrows were taking greater risks than the tits who spent only a short time in the open [50]. Sparrows and finches, however, come to feeders in bigger groups as mixed flocks, which gives them a better chance of noticing potential threats and allows individual birds to spend less time in vigilance (e.g. [46, 47]). Short videos from our experiments to illustrate how birds were visiting feeders next to which were different dummies are available in Additional file 5, Additional file 6, Additional file 7.

### Experimental stimuli

Our experiments compare how birds react to dummies of unmodified dangerous or more or less harmless (apart from size and the resulting advantage in competition at the feeder) birds and to dummies which artificially combine characteristics of dangerous and harmless birds. This allows us to compare the meaning of particular features in the recognition process and to eliminate the effect of competition.

We used soft textile dummies based on the likeness of male Eurasian sparrowhawk, the most dangerous avian predator of small passerines in Europe [17, 51, 52]. The male poses a greater threat to small passerines than the female does [52]. As a control, we used a textile dummy of domestic pigeon, a harmless bird of the same size as the sparrowhawk (32 cm in length). All dummies were made of a wire frame encased in cotton wool wadding and covered in felt. The felt surface was painted with acrylic colours. The beak and talons were made of modelling clay, the eyes were made of glass. This type of dummy has been used in earlier studies that tested the passerines’ ability to recognise predators and the differences between them and taxidermic specimens is only slight [53, 54].

In the following, we present the results of two independent experiments. In the first, we used the following dummies: an unmodified sparrowhawk (hereafter referred to as ‘large hawk’ and abbreviated ‘LH’; Fig. 1a), a downsized sparrowhawk (16 cm long, the size of a great tit; Fig. 1b) with unmodified colouration (‘small hawk’, abbreviated ‘SH’), a life-sized sparrowhawk with pigeon colouration (‘large pigeon-hawk’, abbreviated ‘LPH’; Fig. 1c), a downsized sparrowhawk with pigeon colouration (‘small pigeon-hawk’ SPH; Fig. 1d), and an unmodified pigeon dummy (‘pigeon’, abbreviated ‘P’, Fig. 1e).

In the second experiment, we used the same unmodified dummies of the sparrowhawk and the pigeon (LH, P; Fig. 1a, e) but also life-sized dummies of sparrowhawk with robin and great tit colouration (‘large robin-hawk’, abbreviated ‘LRH’, Fig. 1f; and ‘large tit-hawk’, abbreviated ‘LTH’, Fig. 1g).

### Experimental design

Before presenting each pair of dummies, we recorded 30 min of undisturbed bird visits at both feeders without any dummies in their vicinity. The presentation of one pair of dummies immediately followed this control and took another 30 min: we displayed two dummies at the same time, each on a rod app. 75 cm long, at each of the two feeders. In most trials, one of the dummies was the either the unmodified sparrowhawk dummy (a dangerous bird—dangerous control) or the unmodified pigeon dummy (a harmless bird of comparable size to the sparrowhawk—harmless control). Pigeons do not threaten the tested bird species by predation but may compete with them for food. When a feeder is occupied by numerous birds, the larger-bodied species can push out the smaller ones [49]. We assumed that only dummies whose presence lowers bird attendance at the feeder significantly more than a pigeon dummy does are perceived by birds as more threatening than a comparably sized food competitor. The control dummy of a harmless bird thus allowed us to filter out the effect of food competition among birds at feeders. The other dummy presented together with the unmodified sparrowhawk or pigeon dummy was one of the modified sparrowhawk dummies mentioned above (see Table 6). In addition to experiments with harmful or harmless control dummies, we also compared two downsized dummies (SPH and SH), two of dummies with modified colouration (LTH and LRH), and the two control dummies (P and LH) against each other.

**Table 6:** The schedule of dummies presented in particular trials and the number of series conducted for each combination

Experiments	Focal dummy	Non-focal dummy	Number of series conducted
First	Large hawk (LH)	Pigeon (P)	15
	Large hawk (LH)	Large pigeon-hawk (LPH)	15
	Pigeon (P)	Large pigeon-hawk (LPH)	15
	Large hawk (LH)	Small hawk (SH)	15
	Pigeon (P)	Small hawk (SH)	15
	Large hawk (LH)	Small pigeon-hawk (SPH)	15
	Pigeon (P)	Small pigeon-hawk (SPH)	15
	Small hawk (SH)	Small pigeon-hawk (SPH)	15
	Large pigeon-hawk (LPH)	Small pigeon-hawk (SPH)	15
Second	Large hawk (LH)	Pigeon (P)	15
	Large hawk (LH)	Large tit-hawk (LTH)	15
	Pigeon (P)	Large tit-hawk (LTH)	15
	Large hawk (LH)	Large robin-hawk (LRH)	15
	Pigeon (P)	Large robin-hawk (LRH)	15
	Large tit-hawk (LTH)	Large robin-hawk (LRH)	15

Dummies were placed at the outer corner of the feeder frame so as to face feeder centre. Panasonic HC-V510 cameras were placed at least 15 m away from feeders to record the number of birds visiting the feeders. Experimental days started one hour after dawn. We recorded the presence of snow cover and air temperature. The first experiment was conducted in winters of 2015/2016 and 2016/2017, consisted of 15 repetitions of nine dummy pair presentations (135 trials in total). We recorded 48,647 birds landing at feeders during these trials and 78,060 birds landing at feeders during the preceding empty controls. The second experiment was conducted in the winter of 2017/2018 and consisted of 15 repetitions of six dummy pair presentations (90 trials in total). In this experiment, we recorded 7,451 birds landing at feeders during trials and 27,264 birds landing at feeders during the preceding empty controls. Repetitions of nine (or six) pairs of dummies spanned over two days and the sequence of trials was randomised.

Tested birds were not marked, which means that the composition of visiting birds was anonymous. Nevertheless, we can assume that the effect of learning or habituation was not significant because it has repeatedly been shown in field experiments that the turnover of a bird community visiting feeders is high [15, 40].

### Statistical analyses

As a proxy for fear elicited by each presented dummy, we counted the total number of birds visiting a feeder. Moreover, before each dummy presentation we counted the number of visits to a feeder without a dummy (‘empty feeder’) and used it as a measure of feeder visiting rate. Temporal bias caused by rapid and unpredictable changes in the assemblage of birds in feeder vicinity could be a weakness of feeder experiments such as ours [55–57]. This is why we related the number of visits to a feeder with a dummy to the number of visits to feeder without a dummy immediately before dummy placement. All further analyses then worked with the ratio between the two numbers. Whenever the dummy had a repellent effect, the ratio was below one. We chose this procedure also because it can be assumed that the number of visits can be affected by all dummies (especially a harmless but large pigeon) and we need to know the magnitude of this effect to interpret the results. We transformed these data by arcsine transformation to meet the Gaussian distribution of residuals. In addition, we tested differences in the absolute number of visits to both feeders with dummies. The results are available in Additional file 3 (Table 7, 8).

We obtained the number of visits to the two feeders in each trial simultaneously, so each trial figures in the analyses twice: each feeder was once treated as focal and once as non-focal. The number of visits to the focal and non-focal feeder acted as independent variables. Accordingly, dummy IDs presented at the focal and non-focal feeder were also entered into analyses twice: the same dummy appeared once at the focal feeder (focal dummy) and once at the non-focal feeder (non-focal dummy). To assess the effect of tested predictors on the variability of bird visiting rate to the feeder, we ran linear mixed effects models (LMMs). Because we could not treat a particular trial as independent, the ID of the series (presentation of all nine or six pairs of dummies) was included in the model as a random factor.

The tested predictors were: focal dummy, non-focal dummy, interaction of these two predictors, the birds species, interaction between visiting bird species and the dummy presented at the focal feeder, dummy preceding the currently assessed trial, feeder identity (one of the two actual feeder spots), the order of trials, air temperature on the day of the experiment, and the presence of snow in feeder vicinity.

These predictors were successively added to an empty model that included only the random factor (forward stepwise selection). Models including particular predictors were compared using the likelihood ratio test following Gaussian distribution (chi-squared test). To compare the levels of categorical predictors, we used Tukey HSD post hoc tests (t-test) with Tukey correction for repeated measures. Further, we also used the non-parametric Mann–Whitney test (U test) with Bonferroni correction for pairwise comparisons, which allowed us to decide whether some quantitative insignificant differences can be treated as biologically relevant. All computations were performed in R for Windows software (version R 3.4.4) [58].

## Supplementary Information

**Additional file 1**. Material Table S1 containing analysed data from the first experiment.

**Additional file 2**. Material Table S2 containing analysed data from the second experiment.

**Additional file 3**. Additional Statistical Tests containing Table 7 and 8.

**Additional file 4**. A scheme of the experimental site.

**Additional file 5**. Short video to illustrate how birds were visiting feeders during empty control.

**Additional file 6**. Short video to illustrate how birds were visiting feeders with a pigeon dummy.

**Additional file 7**. Short video to illustrate how birds were visiting feeders  with a sparrowhawk dummy.

## Data Availability

All data generated or analysed during this study are included in this published article (Additional file 1: Table S1, Additional file 2: S2, Additional file 3: Additional Statistical Tests).

## Abbreviations

LH: Unmodified sparrowhawk (‘large hawk’)
P: Unmodified domestic pigeon (‘pigeon’)
SH: Downsized sparrowhawk with unmodified colouration (‘small hawk’)
LPH: Life-sized sparrowhawk with pigeon colouration (‘large pigeon-hawk’)
SPH: Downsized sparrowhawk with pigeon colouration (‘small pigeon-hawk’)
LTH: Life-sized sparrowhawk with great tit colouration (‘large tit-hawk’)
LRH: Life-sized sparrowhawk with robin colouration (‘large robin-hawk’)

## References

[1] Curio E, Farner DS (1976). The ethology of predation. The series zoophysiology and ecology.

[2] Cohen H, Lefebvre C (2017). Handbook of categorization in cognitive science.

[3] Götmark F (2002). Predation by sparrowhawks favours early breeding and small broods in great tits. Oecologia.

[4] Lorenz K (1937). The companion in the bird's world. Auk.

[5] Krätzig H (1940). Untersuchungen zur Lebensweise des Moorschneehuhns (*Lagopus l. lagopus* L.) während der Jugendentwicklung. J Ornithol..

[6] Tinbergen N (1948). Social releasers and the experimental method required fortheir study. Wilson Bull.

[7] Curio E (1975). The functional organization of anti-predator behaviour in the pied flycatcher: a study of avian visual perception. Anim Behav.

[8] Smith JM, Graves HB (1978). Some factors influencing mobbing behaviour in barn swallows *Hirundo rustica*. Behav Biol.

[9] Gill SA, Neudorf DL, Sealy SG (1997). Host responses to cowbirds near the nest: cues for recognition. Anim Behav.

[10] Gill SA, Grieef PM, Staib LM, Sealy SG (1997). Does nest defence deter or facilitate cowbird parasitism? A test of the nesting-cue hypothesis. Ethology.

[11] Edwards G, Hosking E, Smith S (1950). Reactions of some passerine birds to a stuffed cuckoo. II. A detailed study of the Willow Warbler. Br Birds..

[12] Tvardíková K, Fuchs R (2011). Do birds behave according to dynamic risk assessment theory?. A feeder experiment Behav Ecol Sociobiol.

[13] Tvardíková K, Fuchs R (2012). Tits recognize the potential dangers of predators and harmless birds in feeder experiments. J Ethol.

[14] Strnad M, Němec M, Veselý P, Fuchs R (2012). Red-backed Shrikes (*Lanius collurio*) adjust the mobbing intensity, but not mobbing frequency, by assessing the potential threat to themselves from different predators. Ornis Fennica.

[15] Veselý P, Buršíková M, Fuchs R (2016). Birds at the winter feeder do not recognize an artificially coloured predator. Ethology.

[16] Götmark F, Post PG (1996). Prey selection by sparrowhawks, Accipiter nisus: relative predation risk for breeding passerine birds in relation to their size, ecology and behaviour. Philos Trans R Soc Lond, B, Biol Sci.

[17] Chamberlain DE, Glue Chamberalin DE, Toms MP (2009). Sparrowhawk *Accipiter nisus* presence and winter bird abundance. J Ornithol.

[18] Fuchs R, Veselý P, Nácarová J. Predator recognition in birds: the use of key features. Cham: Springer; 2019. https://doi.org/10.1007/978-3-030-12404-5

[19] Scaife M (1976). The response to eye-like shapes by birds. I. The effect of context: a predator and a strange bird. Anim Behav..

[20] Trnka A, Prokop P, Grim T (2012). Uncovering dangerous cheats: how do avianhosts recognize adult brood parasites?. PLoS ONE.

[21] Beránková J, Veselý P, Sýkorová J, Fuchs R (2014). The role of key features in predator recognition by untrained birds. Anim Cogn.

[22] Davies NB, Welbergen JA (2008). Cuckoo–hawk mimicry? An experimental test. P Roy Soc B-Biol Sci.

[23] Swaisgood RR, Owings DH, Rowe MP (1999). Conflict and assessment in a predator–prey system: ground squirrels versus rattlesnakes. Anim Behav.

[24] Templeton CN, Greene E, Davis K (2005). Allometry of alarm calls: black-capped chickadees encode information about predator size. Science.

[25] Palleroni A, Hauser M, Marler P (2005). Do responses of galliform birds vary adaptively with predator size?. Anim Cogn.

[26] Beránková J, Veselý P, Fuchs R (2015). The role of body size in predator recognition by untrained birds. Behav Process.

[27] Quinn JL, Cole EF, Bates J, Payne RW, Cresswell W (2012). Personality predicts individual responsiveness to the risks of starvation and predation. Proc Royal Soc B.

[28] Nácarová J, Veselý P, Fuchs R (2018). Effect of the exploratory behaviour on a bird's ability to categorize a predator. Behav Process.

[29] Soard CM, Ritchison G (2009). Chick-a-dee calls of Carolina chickadees convey information about degree of threat posed by avian predators. Anim Behav.

[30] Courter JR, Ritchison G (2010). Alarm calls of tufted titmice convey information about predator size and threat. Behav Ecol.

[31] Klump GM, Curio E (1983). Reactions of blue tits *Parus caeruleus* to hawk models of different sizes. Bird Behav.

[32] Evans C, Macedonia J, Marler P (1993). Effects of apparent size and speed on the response of chickens, *Gallus-Gallus*, to computer-generated simulations of aerial predators. Anim Behav.

[33] Towe AL (1954). A study of figural equivalence in the pigeon. J Comp Physiol Psychol.

[34] Jenkins WO, Pascal GR, Walker RW (1958). Deprivation and generalization. J Exp Psychol..

[35] Wildemann DG, Holland JG (1973). The effect of the blackout method on acquisition and gene-ralization. J Exp Enal Behav.

[36] Pisacreta R, Potter C, Lefave P (1984). Matching of varying-size form stimuli in the pigeon. Bull Psychon Rev.

[37] Peissig JJ, Kirkpatrick K, Young ME, Wasserman EE (2006). Biederman I Effects of varying stimulus size on object recognition in pigeons. J Exp Psychol Anim Learn Cogn.

[38] Vyazovska OV, Teng Y, Wasserman EA (2014). Attentional tradeoffs in the pigeon. J Exp Anal Behav.

[39] Welbergen JA, Davies NB (2011). A parasite in Wolf's clothing: hawk mimicry reduces mobbing of cuckoos by hosts. Behav Ecol.

[40] Tvardíková K, Fuchs R (2010). Tits use amodal completion in predator recognition: a field experiment. Anim Cogn.

[41] Nováková N, Veselý P, Fuchs R (2017). Object categorization by wild ranging birds—winter feeder experiments. Behav Process.

[42] Grim T (2005). Host recognition of brood parasites: implications for methodology in studies of enemy recognition. Auk.

[43] Jenni L, Kéry M (2003). Timing of autumn bird migration under climate change: advances in long–distance migrants, delays in short–distance migrants. Proc Royal Soc B.

[44] Cepák J, Klvaňa P, Škopek J, Schröpfer L, Jelínek M, Hořák D, Formánek F, Zárybnický J (2008). Atlas migrace ptáků České republiky a Slovenska.

[45] Visser ME, Perdeck AC, van Balen JH, Both C (2009). Climate change leads to decreasing bird migration distances. Glob Change Biol..

[46] Powell GVN (1974). Experimental analysis of the social value of flocking by starlings (Sturnus vulgaris) in relation to predation and foraging. Anim Behav.

[47] Elgar MA, Burren PJ, Posen M (1984). Vigilance and perception of flock size in foraging House Sparrows (*Passer domesticus* L.). Behav..

[48] Newton I (1967). The adaptive radiation and feeding ecology of some British finches. Ibis.

[49] Morse DH (1970). Ecological aspects of some mixed-species foraging flocks of birds. Ecol Monogr.

[50] Moreno E, Carrascal LM (1991). Patch residence time and vigilance in birds foraging at feeders. Implications of bill shape. Ethol Ecolog Evol..

[51] Zawadzka D, Zawadzki J (2001). Breeding populations and diets of the Sparrowhawk *Accipiter nisus* and the Hobby *Falco subbuteo* in the Wigry National Park (NE Poland). Acta Ornithol.

[52] Bujoczek M, Ciach M (2009). Seasonal changes in the avian diet of breeding sparrowhawks *Accipiter nisus*: how to fulfill the offspring's food demands. Zool Stud.

[53] Němec M, Syrová M, Dokoupilová L, Veselý P, Šmilauer P, Landová E, Lišková S, Fuchs R (2015). Surface texture and priming play important roles in predator recognition by the red-backed shrike in field experiments. Anim Cogn.

[54] Nováková N, Veselý P, Fuchs R (2020). Object categorization by wild-ranging birds in nest defence. Anim Cogn.

[55] Austin GT, Smith EL (1972). Winter foraging ecology of mixed insectivorous bird flocks in oak woodland in southern Arizona. Condor.

[56] Fuller RA, Warren PH, Armsworth PR, Barbosa O, Gaston KJ (2008). Garden bird feeding predicts the structure of urban avian assemblages. Divers Distrib.

[57] Wilson WE (2001). The effects of supplemental feeding on wintering black-capped chickadees (*Poecile atricapilla*) in central Maine: population and individual responses. Wilson J Ornithol.

[58] Team RC. R: a language and environment for statistical computing. 2013.

