# Supplementary material for: Untrained birds’ ability to recognise predators with changed body size and colouration in a field experiment
Source: BMC Ecol Evol. 2021 May 1;21:74. doi: 10.1186/s12862-021-01807-8 (PMC8088688; doi:10.1186/s12862-021-01807-8)
Supplement: Supplementary file 3 — Additional file 3. Additional Statistical Tests containing Table 7 and 8. [file 12862_2021_1807_MOESM3_ESM.docx]

Supplemental Material S3: Additional statistical tests

## Nonparametric pairwise post hoc comparison (Mann–Whitney U test) of the proportion of visits to feeders with particular dummies in the first and second experiment.

When using a nonparametric M–W test instead of Tukey HSD test in post hoc comparisons of the proportion of visits particular dummies, we obtained – with one exception – identical results (Tab. 7).

**Tab. 7** Results of nonparametric pairwise post hoc comparisons (Mann–Whitney U tests) of the proportion of visits to feeders with particular dummies in the first experiment.

| Dummy | LH | SH | LPH | SPH | P |
| --- | --- | --- | --- | --- | --- |
| LH | - | **-4.931** | **-4.122** | **-5.540** | **-3.602** |
| SH | **<0.001** | - | - | -1.948 | **3.912** |
| LPH | **<0.001** | - | - | **-4.506** | -1.410 |
| SPH | **<< 0.001** | 0.051 | **< 0.001** | - | **4.207** |
| P | **< 0.001** | **< 0.001** | 0.1586 | **< 0.001** | - |

*The right-top part of the table refers to Z-values, the left-bottom part to p-values.* *Positivity of Z-values indicates that the number of visits to feeder with dummy in a column is larger than the number of visits to feeder with dummy in the row. LH = unmodified sparrowhawk (‘large hawk’); P = unmodified domestic pigeon (‘pigeon’); SH = downsized sparrowhawk with unmodified colouration (‘small hawk’); LPH = life-sized sparrowhawk with pigeon colouration (‘large pigeon-hawk’); SPH = downsized sparrowhawk with pigeon colouration (‘small pigeon-hawk’). Significant effects are in bold.*

The relative decrease in the number of visits to feeder with the downsized sparrowhawk dummy with unmodified colouration was greater than the relative decrease in the number of visits to feeder with the downsized pigeon-coloured sparrowhawk dummy (Tab. 7).

In the second experiment – in contrast to the first experiment – nonparametric M–W post hoc test yielded results of a lower level of significance. We can see that the relative decrease in the number of visits to feeder with the unmodified sparrowhawk dummy and that with unmodified pigeon dummy did not differ (Tab. 8).

**Tab. 8** Results of nonparametric pairwise post hoc comparisons (Mann–Whitney U tests) of the proportion of visits to feeders with particular dummies in the second experiment.

| Dummy | LH | LTH | LRH | P |
| --- | --- | --- | --- | --- |
| LH | - | **-2.081** | -1.896 | -1.631 |
| LTH | **0.037** | - | -0.063 | -1.928 |
| LRH | 0.076 | 0.950 | - | **-2.256** |
| P | 0.103 | 0.054 | **0.013** | - |

*The right-top part of the table refers to Z-values, the left-bottom part to p-values. Positivity of Z-values indicates that the number of visits to feeder with dummy in a column is larger than the number of visits to feeder with dummy in the row. LH = unmodified sparrowhawk (‘large hawk’); P = unmodified domestic pigeon (‘pigeon’); SH = downsized sparrowhawk with unmodified colouration (‘small hawk’); LPH = life-sized sparrowhawk with pigeon colouration (‘large pigeon-hawk’); SPH = downsized sparrowhawk with pigeon colouration (‘small pigeon-hawk’). Significant effects are in bold.*

The relative decrease in the number of visits to feeder with the life-sized robin-coloured sparrowhawk dummy was slightly lower than the relative decrease in the number of visits to feeder with the unmodified sparrowhawk dummy. The relative decrease in the number of visits to feeder with the life-sized great tit-coloured sparrowhawk dummy was slightly higher than the relative decrease in the number of visits to feeder with the unmodified pigeon dummy (Tab. 8).

## T-test of the absolute numbers of visits to feeders with particular dummies in the first and second experiment.

To verify our results, we compared the absolute numbers of visits instead of the visit ratio to both feeders with simultaneously presented dummies.

We compared the number of birds visiting the focal and the non-focal feeder with the expected random numbers of visits to feeders with both dummies (sum of the number of birds visiting both feeders divided by two, which is the number of feeders available at the moment).

**Tab. 9** Comparisons of deviation of the absolute numbers of visits to feeders with particular dummy combinations from chance in the first experiment (T-tests).

| Dummy combination | t-value | p-value |
| --- | --- | --- |
| LH x SH | 3.891 | 0.002 |
| LH x LPH | 2.81 | **0.014** |
| LH x SPH | 2.917 | **0.012** |
| LH x P | 3.254 | **0.006** |
| SH x SPH | 0.386 | 0.706 |
| SH x P | 2.656 | **0.019** |
| LPH x SPH | 2.549 | **0.024** |
| LPH x P | 1.457 | 0.167 |
| SPH x P | 2.451 | **0.028** |

*T-values refer to the magnitude of difference between the mean value of number of visits to feeders with the particular pairs of dummies and chance. LH = unmodified sparrowhawk (‘large hawk’); P = unmodified domestic pigeon (‘pigeon’); SH = downsized sparrowhawk with unmodified colouration (‘small hawk’); LPH = life-sized sparrowhawk with pigeon colouration (‘large pigeon-hawk’); SPH = downsized sparrowhawk with pigeon colouration (‘small pigeon-hawk’). Significant effects are in bold.*

**Tab. 10** Comparisons of deviation of the absolute numbers of visits to feeders with particular dummy combinations from chance in the second experiment (T-tests).

| Dummy combination | t-value | p-value |
| --- | --- | --- |
| LH x LTH | 3.317 | **0.005** |
| LH x LRH | 1.468 | 0.166 |
| LH x P | 2.639 | **0.019** |
| LTH x LRH | 0.574 | 0.575 |
| LTH x P | 0.843 | 0.414 |
| LRH x P | 3.275 | **0.006** |

*T-values refer to the magnitude of difference between the mean value of the number of visits to feeders with particular pairs of dummies and chance. LH = unmodified sparrowhawk (‘large hawk’); P = unmodified domestic pigeon (‘pigeon’); SH = downsized sparrowhawk with unmodified colouration (‘small hawk’); LPH = life-sized sparrowhawk with pigeon colouration (‘large pigeon-hawk’); SPH = downsized sparrowhawk with pigeon colouration (‘small pigeon-hawk’). Significant effects are in bold.*

The results of comparisons between the absolute numbers of visits and chance in both experiments are highly similar to the proportion of landing birds. They slightly differ in the degree of significance.
