# Supplementary figures and images for: Untrained birds’ ability to recognise predators with changed body size and colouration in a field experiment
Source: BMC Ecol Evol. 2021 May 1;21:74. doi: 10.1186/s12862-021-01807-8 (PMC8088688; doi:10.1186/s12862-021-01807-8)

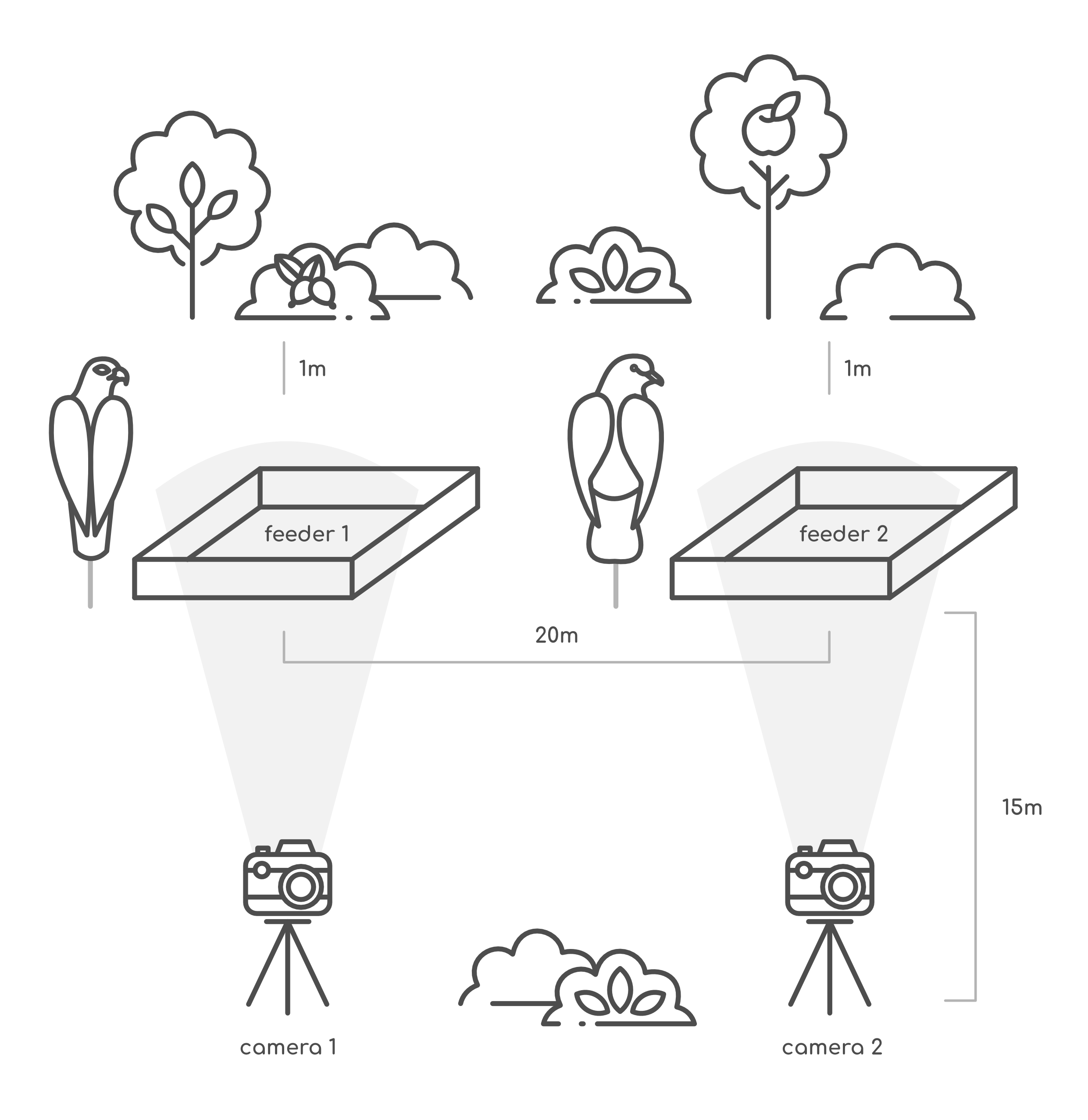

Supplement: Supplementary file 4 — Additional file 4. A scheme of the experimental site. [file 12862_2021_1807_MOESM4_ESM.tiff]
